# Prion propagation and cellular dysfunction in prion disease: Disconnecting the dots

**DOI:** 10.1371/journal.ppat.1011714

**Published:** 2023-10-26

**Authors:** Simote T. Foliaki, Cathryn L. Haigh

**Affiliations:** Laboratory of Neurological Infections and Immunity, Division of Intramural Research, Rocky Mountain Laboratories, National Institutes of Health, Hamilton, Montana, United States of America; Stanford University, UNITED STATES

## Introduction

Prion diseases are rare and incurable neurodegenerative diseases caused by misfolding of prion protein (PrP) into abnormal disease-associated isoforms (PrP^D^), which accumulate into insoluble aggregates. In humans, PrP^D^ are formed by sporadic events, genetic mutations within the *PRNP* gene that encodes PrP, or exposure to exogenous PrP^D^. The clinical disease includes a spectrum of neurological phenotypes ranging from cognitive impairment to severe ataxia. The detection of PrP^D^ in the central nervous system (CNS) distinguishes prion disease from other clinically related disorders. Due to the broad range of symptoms and rapid progression, diagnosis often happens late in the disease or at death, thus increasing the correlation between PrP^D^ detection and clinical disease. A similar correlation is observed in animal models of prion disease where increased PrP^D^ are detected as disease progresses [1]. In these models, the increasing detection of PrP^D^ is linked to neuronal cytoskeletal breakdown and loss, suggesting that PrP^D^ are directly responsible for neurodegeneration. This has led to numerous mouse studies demonstrating that PrP^D^ are neurotoxic in that they directly cause dendritic spine pruning and synaptic loss that disrupt neuronal network communication [2,3]. However, these neurotoxic phenotypes are only significantly evident in clinical disease, whereas the detection of transmissible PrP^D^ is possible in asymptomatic mice [1]. This has led to the two-phase hypothesis proposing that there is an exponential propagation of transmissible PrP^D^ earlier in the disease, followed by a sudden production of neurotoxic PrP^D^ at late-stage disease, correlating with the detection of neurotoxic phenotypes and clinical onset [1]. While this theory reveals how PrP^D^ evolve during disease, it does not consider the contribution of cellular and molecular homeostasis to disease pathogenesis.

Much discussion in the past has focused on loss or gain/corruption of PrP function as contributing to the neurotoxic phenotype, but through human and animal models of prion disease-causing mutations, we can disconnect some of the PrP functional changes from those changes that are specifically caused by the misfolded species associated with propagation. The secondary and tertiary structure of the misfolded prion is thought to be the governing determinant of disease phenotype; however, evidence exists, through these disease-causing mutations, that some aspects are encoded in the primary sequence of the protein with a single amino acid change sufficient for the phenotype to emerge. Here, we will discuss the relationships between PrP mutations and altered neuronal function, astrogliosis, and cellular and molecular homeostatic dysfunction in the absence of PrP^D^.

## Mutation alone is not sufficient to cause infectious disease

Various mouse models have been developed to express either mouse or human PrP containing mutations associated with human genetic prion diseases [4]. A number of these transgenic (Tg) mouse models, especially those expressing the mutations within human PrP, develop clinical diseases but do not produce PrP^D^ that are transmissible [4,5]. Models lacking transmissible PrP^D^ include the Fatal Familial Insomnia (Tg(FFI)) D177N mutation with methionine at codon 128 (D177N/M128) [5,6], Gerstmann-Sträussler-Scheinker syndrome (GSS) P102L mutation [7], genetic Creutzfeldt Jakob Disease (gCJD) D177N mutation with valine at codon 128 (D177N/V128; Tg(CJD)) [5,8], gCJD with a 14-octapeptide insertion (Tg(PG14)) [5,9], and the gCJD E200K mutation [7]. Other mouse models, mostly those expressing mutant mouse or bank vole PrP, developed infectious disease [4], supporting that both the PrP species and the rodent background influences spontaneous disease and the propagation of infectious prions.

To address the role of mutation in a completely human background, our group and others have developed human cerebral organoid models of genetic prion diseases from human induced pluripotent stem cells. The PrP mutations were either carried by the donors or introduced by CRISPR-cas9 cloning. The mutations in these models include D178N/M129 (FFI) [10], E200K [11,12], an 8-octarepeat insertion (gCJD; [13]), and Y218N (GSS; [14]). Cerebral organoids are a new model of human brain tissue. They are small spheres of neuronal lineage cells, including neurons, oligodendrocytes, and astrocytes, which self-organize into a 3D brain-like structure, resembling some aspects of a complex brain [15]. To date, none of the disease-associated mutations modeled in human cerebral organoids or spherical masses of neurons have been shown to produce a spontaneous infection. Furthermore, no spontaneous PrP^D^ production was detected in E200K organoids subjected to oxidative stress or viral insults [16]. Despite the lack of prion disease hallmarks such as PrP^D^ accumulation and spongiosis, significant cellular and molecular dysfunctions or damages are found in the *PRNP* E200K, D178N, and Y218N mutation organoids [10,11,14].

## Mutation is sufficient to cause neuronal dysfunction and change synaptic composition

Investigation of 2 highly penetrant genetic prion diseases, the E200K gCJD and D178N FFI in human cerebral organoid models revealed neuronal network dysfunction [10,12]. The unaffected control, E200K and D178N organoids all underwent normal maturation processes with their neuronal network connectivity becoming stronger and more complex between 3 and 6 months old. After 6 months old, the E200K organoids displayed suppressed activity compared with control organoids, and further investigation showed altered synaptic composition, shifting their excitatory and inhibitory balance toward the former [12]. Gamma-aminobutyric acid (GABA) detection and the GABAergic system were suppressed in both E200K and D178N/M129 organoids, but increased acetylcholine was observed in the D178N organoids [10,12]. In the Tg(FFI) and Tg(CJD), and Tg(PG14) mice, similar neuronal dysfunction disrupts sleep, motor function, cognition, and memory despite the lack of prion infectivity [5,6,8,17]. Given the mutations change PrP conformation, it is likely that some of these neuronal dysfunctions reflect a loss of PrP function as coisogenic PrP knockout (KO) mice have altered neuronal function [14].

That PrP can be responsible for transducing neuronal dysfunction in the absence of propagating prions is in alignment with a different approach to studying prion toxic signalling. Toxic signalling mediated by the N-terminus of PrP can be stimulated using ligands, including antibody fragments that target the C-terminal globular domain of PrP within the first and third alpha helicies [18]. Neurotoxicity was linked with spontaneous ionic currents that could be recorded by patch clamping and with degeneration of dendrites [19]. Control of the N-terminal region by the C-terminus may be related to the copper-binding properties of full-length PrP, which could be altered by the presence of some of the disease-associated mutations [20]. Furthermore, similar spontaneous ionic currents have been observed in cultured cells transfected with several PrP disease-associated mutations [21]. Altogether, these studies support that changes within the prion protein caused by the mutations alone are sufficient to exert an effect on neurotransmission that is independent of propagation and likely reflects a corruption of PrP function.

## Astrogliosis occurs in the presence of PrP mutations

A common hallmark of prion disease pathology is increased reactive astrocytes or astrogliosis [1]. Human neural cultures of Y218N cells showed a significant increase in GFAP mRNA with age, which was attributed to hypertrophic reactive astroglial cells [14]. D178N FFI organoids also demonstrated increased astrogliosis [10], and FFI mice, likewise, exhibited astrogliosis in the absence of propagating prions [6]. The lack of prion propagation disconnects astrogliosis from propagating prions but does not separate astrocytes from disease toxicity, as these cells are known to exist in many activation states within the brain [22]. Further, the Tg(gCJD) mouse model (D177N/V128) exhibited increased astrogliosis linked to neuronal dysfunction and clinical disease [8], supporting the astrocyte-related toxicity without propagating prions.

## Energy balance is perturbed during disease and as a result of PrP mutation

During human prion diseases changes are also seen in brain metabolism including glucose usage and mitochondrial function [23,24]. The D178N FFI organoids showed significantly increased oxidative stress associated with altered bioenergetics, including increased mitochondrial activity [10] but decreased glucose utilization, and caused a general blockage of glycolysis. Human cardiomyocytes with the E200K PrP mutation also showed significantly altered cell metabolism and bioenergetics, indicating the functional impact of PrP mutation is not limited to the brain [25]. Mitochondrial abnormalities have been reported in PrP KO mice [26], and, therefore, this could represent at least a partial loss of PrP function phenotype.

## Cellular damage can occur in the absence of propagating prions

Y218N spherical masses, while showing no propagation, did show 2 other hallmarks of GSS. The neurons showed hyperphosphorylation of Tau and neurofibrillary degeneration [14]. In addition, mitochondria were hyperpolarized in the D178N FFI organoids, indicating damaged mitochondria, which increased autophagy, including mitophagy. This strongly correlated with a significant loss of lipids, including neutral lipids and lipid droplets in these organoids [10]. Further, autophagy also appeared abnormal in the brains of Tg(FFI) mice, where the autophagosomes had anomalous structures with increased lipofuscins, indicating abnormally pigmented lysosomes [6]. Importantly, FFI and gCJD mice lacking transmissible PrP^D^ showed dysfunctional and damaged cellular compartments, such as swollen ER and cisternae of the Golgi complex and abnormal retention of PrP in the ER [6,8,17]. Therefore, we speculate that mutant PrP disrupts cellular compartments, which increases autophagy in order to repair the damage, thus causing a depletion of lipids, as we observed in the D178N FFI organoids [10].

## Conclusions

**Fig 1:**
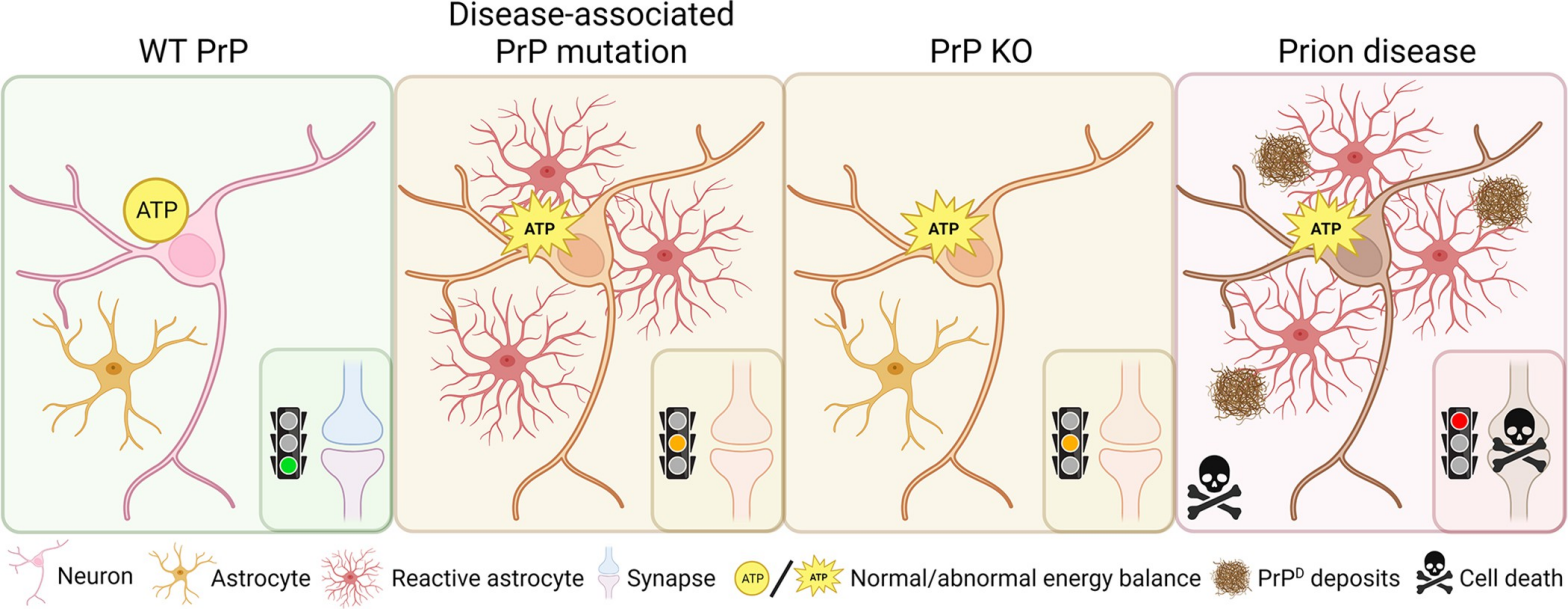
Schematic representation of the changes reported due to *PRNP* mutation, PrP KO, and prion disease. Created with Biorender. KO, knockout; PrP, prion protein; WT, wild-type.

The aspects of prion disease reviewed here, and summarized in Fig 1 with more detail found in Table 1, that can be disconnected from the propagation of infectious prions likely point to a corruption of the PrP function. They may parallel the earliest changes in disease before patients become symptomatic. Detailed interrogation of the preclinical stage in animal models has found cellular abnormalities associated with the early detection of PrP^D^, including free-radical accumulation, increased total PrP, and heightened astrogliosis [27]. Additionally, PrP^D^ produced early during disease in mice have been shown to be transmissible and neurotoxic [3]. Together, these data argue against the two-phase hypothesis as PrP^D^ produced at any stage of disease exert some degree of neurotoxicity. During early disease, this neurotoxicity is less evident as cells appear to have the capacity to compensate. Evidence of such coping mechanisms has been observed in asymptomatic human carriers of PrP mutations, including magnetic resonance diffusion abnormalities [28], elevated neurofilament in cerebrospinal fluid [29], and increased plasma total PrP [30]. The disconnections between disease features and the disease itself highlighted herein indicate that, while the change in conformation causes death as the ultimate outcome of prion diseases, mutations within the primary sequence of natively folded PrP also encode aspects of cellular dysfunction prior to misfolding. These disconnects between prion disease and how disease-associated mutations alter neuronal activity may facilitate future studies determining where benign functional alteration changes into malignant dysfunction during disease and apply this to develop therapeutic interventions that delay clinical onset in genetic prion diseases.

**Table 1:** Characteristics of prion mutations in the absence of propagating prions and prion disease compared with prion disease and PrP knockout (KO).

	Prion disease	FFI-associated mutation		gCJD-associated mutation		GSS-associated mutation		PRNP KO
		Mouse	Human CO	Mouse^1^	Human CO^2^	Mouse^3^	Human CO^4^	Mouse
Misfolded, deposited PrP	✔	✔	X	✔	X	X	X	X
Unusually modified PrP^5^	✔	✔	✔	✔	X	X	X	X
Transmissible PrP	✔	X	X	X	X	X	X	X
Neuronal dysfunction	✔	✔	✔	✔	✔	X	✔	✔
Astrogliosis	✔	✔	✔	✔	Nr	X	✔	X
Energy imbalance	✔	Nr	✔	X/Nr	Nr	Nr	Nr	✔
Cellular damage^6^	✔	✔	✔	X	Nr	X	✔	X
Death from prion disease^7^	✔	X	X	X	X	X	X	X

✔ = present, X = absent, Nr = not reported, CO = cerebral organoid or similar model.

^1^huE200K, msD177N-129V, msPG14 [4,8,9].

^2^huE200K [4,7].

^3^huP102L [4,7].

^4^huY218N [14].

^5^Changed glycosylation or fragmentation.

^6^Hyperphosphorylation of Tau; neurofibrillary degeneration; damaged mitochondria, endoplasmic reticulum, and/or Golgi.

^7^Terminal disease in mice or organoid cultures cease to be viable (as measured by cessation of metabolic activity and media LDH release) with evidence of prion propagation.
